# Research on emergency management of global public health emergencies driven by digital technology: A bibliometric analysis

**DOI:** 10.3389/fpubh.2022.1100401

**Published:** 2023-01-11

**Authors:** Chao Wen, Wei Liu, Zhihao He, Chunyan Liu

**Affiliations:** ^1^School of Emergency Management, Xihua University, Chengdu, China; ^2^College of Management Science, Chengdu University of Technology, Chengdu, China; ^3^School of Automation and Electrical Engineering, Chengdu Institute of Technology, Chengdu, China

**Keywords:** digital technology, public health, emergency management, bibliometrics, big data, machine learning

## Abstract

**Background:**

The frequent occurrence of major public health emergencies globally poses a threat to people's life, health, and safety, and the convergence development of digital technology is very effective and necessary to cope with the outbreak and transmission control of public epidemics such as COVID-19, which is essential to improve the emergency management capability of global public health emergencies.

**Methods:**

The published literatures in the Web of Science Core Collection database from 2003 to 2022 were utilized to analyze the contribution and collaboration of the authors, institutions, and countries, keyword co-occurrence analysis, and research frontier identification using the CiteSpace, VOSviewer, and COOC software.

**Results:**

The results are shown as follows: (1) Relevant research can be divided into growth and development period and rapid development period, and the total publications show exponential growth, among which the USA, China, and the United Kingdom are the most occupied countries, but the global authorship cooperation is not close; (2) clustering analysis of high-frequency keyword, all kinds of digital technologies are utilized, ranging from artificial intelligence (AI)-driven machine learning (ML) or deep learning (DL), and focused application big data analytics and blockchain technology enabled the internet of things (IoT) to identify, and diagnose major unexpected public diseases are hot spots for future research; (3) Research frontier identification indicates that data analysis in social media is a frontier issue that must continue to be focused on to advance digital and smart governance of public health events.

**Conclusion:**

This bibliometric study provides unique insights into the role of digital technologies in the emergency management of public health. It provides research guidance for smart emergency management of global public health emergencies.

## 1. Introduction

The COVID-19 infectious pneumonia pandemic has been sweeping the globe, bringing harm to people's life and health, and at the same time, it poses a severe threat to the market economy and even global security and stability (1). Scientific and efficient emergency management can control the spread of the epidemic and minimize the harm of public emergencies. Public health emergency (PHE) is a major infectious dis-ease due to a lack of prediction and effective prevention. It is also a disease of unknown cause to public health in groups, major occupational or food poisoning, or other events that seriously affect public life and property (1). And it is characterized by suddenness, urgency, uncertainty, multiple subjects, and social impact (2–4). The novel coronavirus, first identified in late 2019, is a major public health emergency, and by the end of November 2022, the World Health Organization reported that the total number of new coronavirus pneumonia (COVID-19) cases had exceeded 625 million, with ~15 million direct and indirect deaths due to new coronaviruses (5), which are still spreading globally. In addition to COVID-19, the world has seen several other major public health emergencies in the last decade, including severe acute respiratory syndrome (SARS) in 2003, H1N1 influenza in 2009, Middle East respiratory syndrome (MERS) in 2012, and West Africa Ebola in 2014, all of which had a major impact on human society (6). These diseases seriously endangered the collective survival and harmonious coexistence of the human community of destiny and caused incalculable damage to the global economy.

In the context of the current iterative development of the smart society, digital technology provides an opportunity to improve the effectiveness of emergency management of public health emergencies, and the innovation and application of big data technology promote the traditional emergency management approach toward “smart management,” “cloud management,” and “visual management.” The conventional thinking, mechanism, and technology of emergency management are undergoing constant deconstruction, reconstruction, and dynamic control of construction. In the face of the prevention and control of the new crown pneumonia epidemic, digital technologies such as big data, artificial intelligence, and cloud computing play an important role in epidemic monitoring and analysis, virus tracing, prevention, control and treatment, and resource deployment (7–11). However, in practical applications, there are also many problems in big data governance of public health emergencies. Therefore, how to use digital technology for accurate disease prediction, scientific decision management, and crisis resolution in public health emergencies. It is an essential scientific topic that needs to be studied as soon as possible.

How digital technologies are embedded and driven to improve the effectiveness of emergency management of public health events (12, 13). It is necessary to systematically review and summarize the existing literature to enrich the theoretical and reliable research framework. The functional, algorithmic, learning, and textual properties of digital technologies have made them a hot topic in public health management (14–16). By combing through the existing literature, we can find that emergency management of public health events driven by digital technology is mainly studied from the following perspectives: first, the use of digital technology for early warning and prediction of public health events, for example, some scholars applied big data on social media to predict health-related behaviors to prepare for and respond to public health emergencies promptly and epidemics (17–19); with the development of digital technologies, intelligent algorithms (8, 20), machine learning (21, 22), deep learning (6, 23) have also been used in early warning studies of public health emergencies to extract and analyze relevant news and other information data intelligently. The application of these methods advances the intelligent emergency management of public health emergencies, thus reducing the impact of public health emergencies on society.

Second, digital technologies are utilized to prevent, control, and monitor public health emergencies. Yin et al. (24) integrated the continuous mechanism of big data intelligence innovation into a complex network and constructed a three-dimensional collaborative epidemic prevention model to reveal the effect of constant epidemic prevention under different policy levels of big data intelligence emergency management. Hu et al. (25) summarized three major roles of big data in preventing and controlling public health emergencies: weaving the joint prevention and control network, carrying out multidimensional information tracing, and ensuring the dynamic balance of the deployment and supply of prevention and control materials. Third, digital technology for “risk-prevention-control” response and decision-making for public health emergencies. Zhu et al. (26) used 3S technology, closely related to artificial intelligence, to design and establish a response system for public health emergencies to improve the government's response and decision-making capacity for responding to and handling public health emergencies and reducing the occurrence of emergencies. Li et al. (27) used healthcare big data analysis technology to analyze COVID-19 information empirically and construct an epidemic information public opinion warning model to identify reliable epidemic information and achieve epidemic risk information screening and prevention and control.

In summary, the existing studies provide a rich theoretical foundation for this paper. Summarizing the role and performance of big data technology in the process of public health governance through a multidimensional, multi-scale, and holistic perspective can help enrich the theory of emergency management of public health emergencies while enhancing the intelligent system response of emergency management system of public health events (13, 24). However, there are few literature reviews on digital technology development status and trends in managing public health emergencies at this stage. The development trend of public health emergency management toward intelligence and digitalization driven by digital technology and its deeper operational logic has not been systematically summarized (28–30). Therefore, this paper uses a bibliometric approach to study the application of digital technology in the emergency management of public health emergencies, focusing on the following issues.

(1) Who are the collaborating countries, collaborating institutions, and collaborating authors on digital technology-driven emergency management for public health emergencies, and how do they contribute to the development of this research?
(2) What are the research hotspots and trends in digital technology-driven emergency management for public health emergencies?
(3) What are the changes in emergency management of public health emergencies driven by digital technology, and what are the future research frontiers?

## 2. Methodology

### 2.1. Data sources and preprocessing

**Figure 1:**
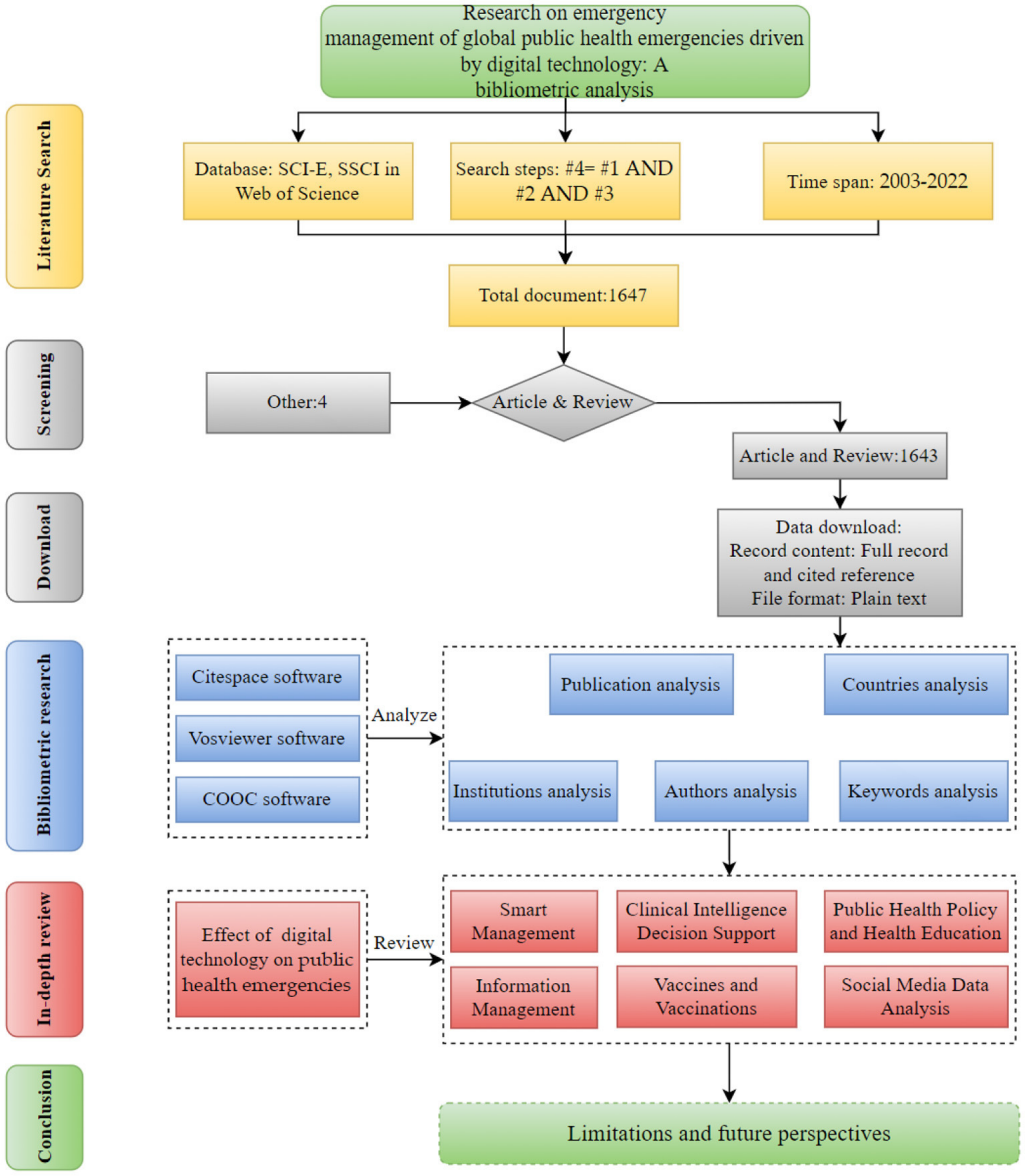
The structural flow diagram. #1={TS=[(“digital techn*” OR “digitiz” OR “big data” OR “data-driven” OR “data driv*” “cloud comput*” OR “internet of thing*” OR “IOT” OR “blockchain” OR “AI” OR “artificial intelligen*” OR “computer vision” OR “machine vision” OR “machine learning” OR “neural network” OR “bayes” OR “supervis* learn*” OR “reinforc*” OR “Support Vector Machine*” OR “classification trees” OR “random forest” OR “Gaussian process” OR “decision tree” OR “algorith” OR “deep learning” OR “boost*” OR “natural language process“ OR “text mining” OR “text* analysis” OR “text data mining”)]}; #2= TS= (”emergenc*“ OR sudden) AND (“public health” OR “2019-nCOV” OR “2019 novel coronaviru*” OR “2019 novel- Cov” OR “COVID-19*” OR “SARS-CoV-2”) OR (“severe acute respiratory syndrome” OR “SARS-coronavirus” OR “SARS-Cov“) OR (“H1N1 influenza” “swine flu influenza” OR “influenza A H1N1” OR “S-OIV” OR “swin-origin influenza”) OR (“Middle East respiratory syndrome” OR “MERS-CoV” OR “MERS-coronavirus”) OR (“EBHF” OR “Ebola hemorrhagic fever” OR “Ebola virus disease” OR “Ebola epidemic” OR “Ebola virus infection”); #3= [TS= (manage*or govern* or control* or warn* or measure* or guid* or lead* or conduct* or response* or regulat* or strategie* or decision* or predict* or “deal with” or countermeasure* or coping*)]; #4= #1 AND #2 AND #3; * represents any group of characters, including null characters. “digital techn*” denotes digital technology, digital techniques, and other quotation marks with the same meaning.

The most critical part of a review article is the data source, and the quantity and quality of relevant documents directly determine the visualization and quality of the review article (31). In order to better grasp the development status and hotspots of digital technology in the field of emergency management of public health emergencies, this paper selects the Science Citation Index Expanded (SCI-E) and Social Science Citation Index (SSCI) databases in the core database of Web of Science (WOS) as the source of literature search data. This database has the characteristics of authority and massive data, and the Web of Science database is more suitable for building knowledge graphs than other databases (32, 33). The time frame of this paper is 2003–2022. Since this work was completed at the end of October 2020, there should be more relevant literature on digital technologies in emergency management of public health emergencies in 2022 than in this paper. To find relevant and appropriate articles, we followed specific steps to collect and select data (1). First, the core collection of Web of Science databases was selected, and second, advanced search settings were used. Finally, search criteria were set to search, and the final desired literature was filtered. The specific research protocol is shown in Figure 1.

### 2.2. Research method

Bibliometrics is a quantitative analysis method that deals with data and information visualization (34). It employs mathematical and statistical methods to reveal research frontiers and hotspots and track trends in a field (35). It is also widely utilized in public health emergency research (36, 37). Currently, visualization tools commonly applied to bibliometric analysis include Citespace (38), VOSviewer (39), and Bibliometrix (40). CiteSpace is often used to analyze data from a large amount of relevant literature on keywords, authors, countries, institutions, and subject categories to predict new trends in a research area (41). VOSviewer is a software that creates maps of the institution, author, and country collaborations or keyword co-occurrence maps based on literature data (42). COOC is an emerging bibliometric software (43). This software can complement the analysis of VOSviewer and CiteSpace by providing additional measurements. In this paper, we combine the advantages of these three software programs. First, we analyzed the major countries, journals, and authors of research on digital technologies in emergency management in the field of public health emergencies using VOSviewer software. Second, we used Citespace and COOC to analyze the current status, hot spots, and frontiers of research on digital technologies in emergency management in public health emergencies.

## 3. Results analysis

### 3.1. Annual distribution and trends of the literature

**Figure 2:**
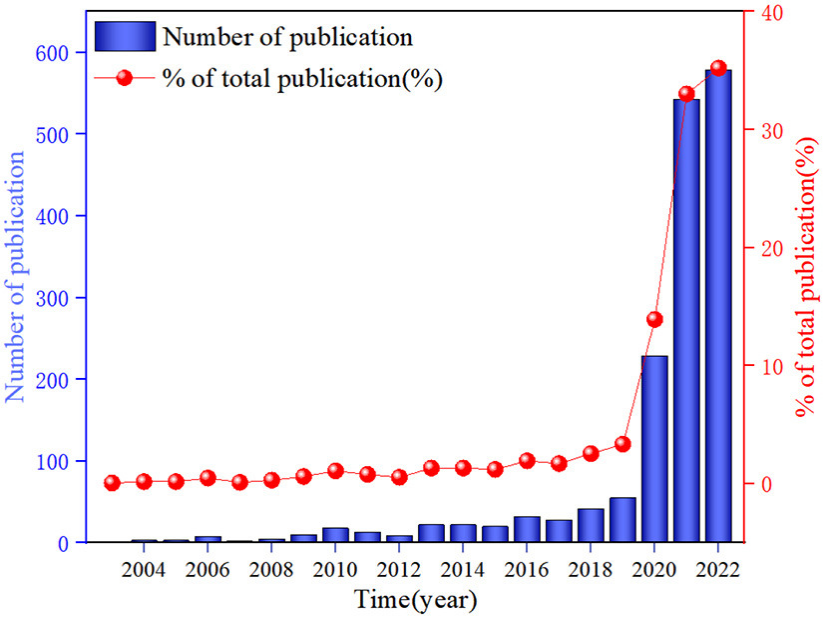
The evolution of publication in emergency management of global public health emergencies driven by digital technology during 2003–2022.

The analysis of the number of published articles provides insight into an academic field trend. Figure 2 shows the annual number of publications related to emergency management of public health emergencies driven by digital technologies and the percentage of cumulative publications. In Figure 2, it can be found that there were not many applications in emergency management of public health emergencies driven by digital technologies. And the number of publications does not exceed 50 before 2019. However, the number of publications reached 229 in 2020, after which the number of published papers showed a continuous increase, mainly since the COVID-19 pandemic caused great concern worldwide. Experts and scholars realized that digital technology plays a vital role in COVID-19 epidemic surveillance and analysis, virus tracing, prevention and control, and treatment and resource deployment.

### 3.2. Cooperation between countries

**Figure 3:**
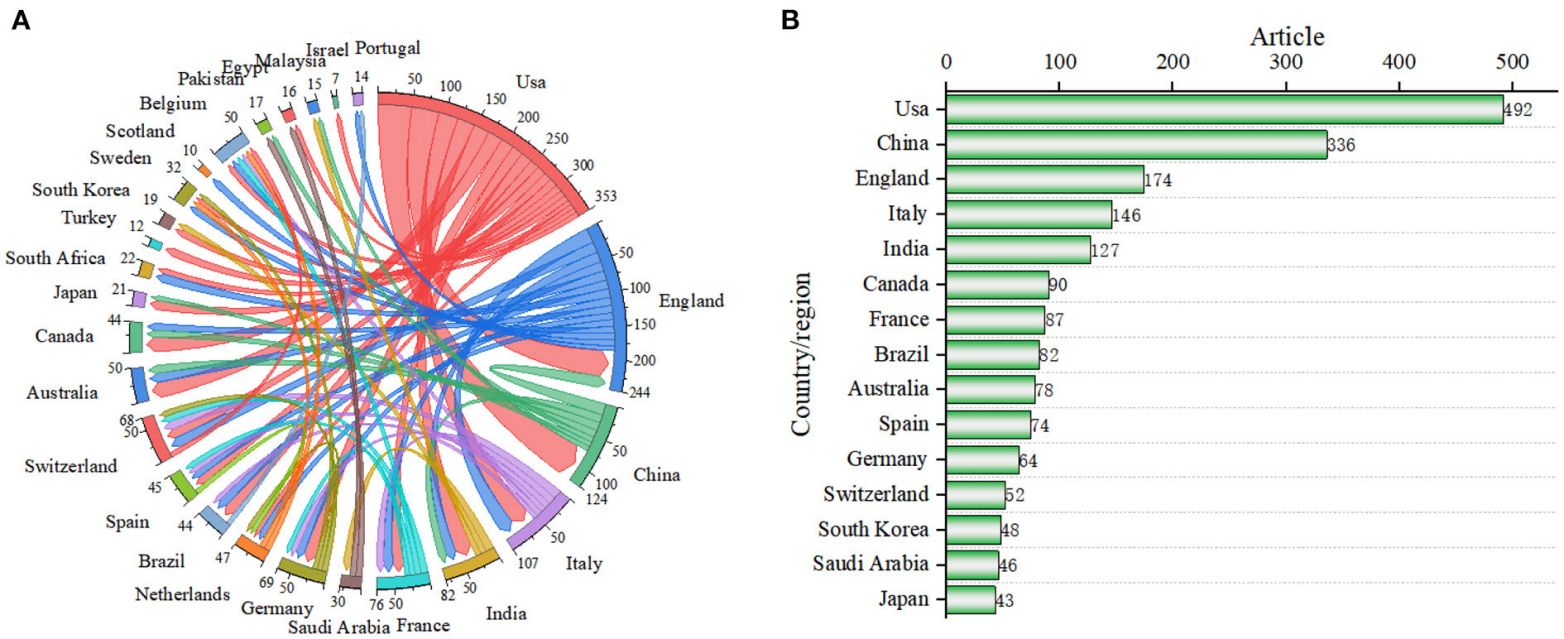
**(A)** Mapping knowledge domains of co-authoring countries/regions. **(B)** Top 15 countries in the number of articles published.

To identify the productive and influential countries/regions for research on emergency management of public health emergencies with digital technologies and the collaboration among them, we analyzed the country/region distribution (Figure 3A). As many as 120 countries and regions are involved in this field (only the top collaborating countries/regions are listed in Figure 3). The top five countries and regions are the USA, China, UK, Italy, and India, with 492 (17.6%), 336 (12.1%), 174 (6.2%), 146 (5.2%), and 127 (4.6%) publications (Figure 3B).

In Figure 3A, the nodes represent different countries or regions. The thickness of the connection between them represents the intensity of cooperation between them—the thicker the linkage, the stronger the cooperation between two countries or regions. The figure shows frequent collaboration between the USA, China, the United Kingdom, Italy, Australia, and Canada.

Based on the strength of the connection, the USA has the highest level of international collaboration, far surpassing other countries/regions. The USA is a world leader in academic research on emergency management of digital technology-driven public health emergencies. Of course, other countries have also made a lot of efforts and cooperation to progress research in this field. The intensity of collaboration among the United Kingdom, China, and Italy follows the United States, indicating that the future trend of digital technology application in public health emergencies also shows the development of multi-country and multi-location diversified cooperation.

### 3.3. Cooperation between institutions

**Figure 4:**
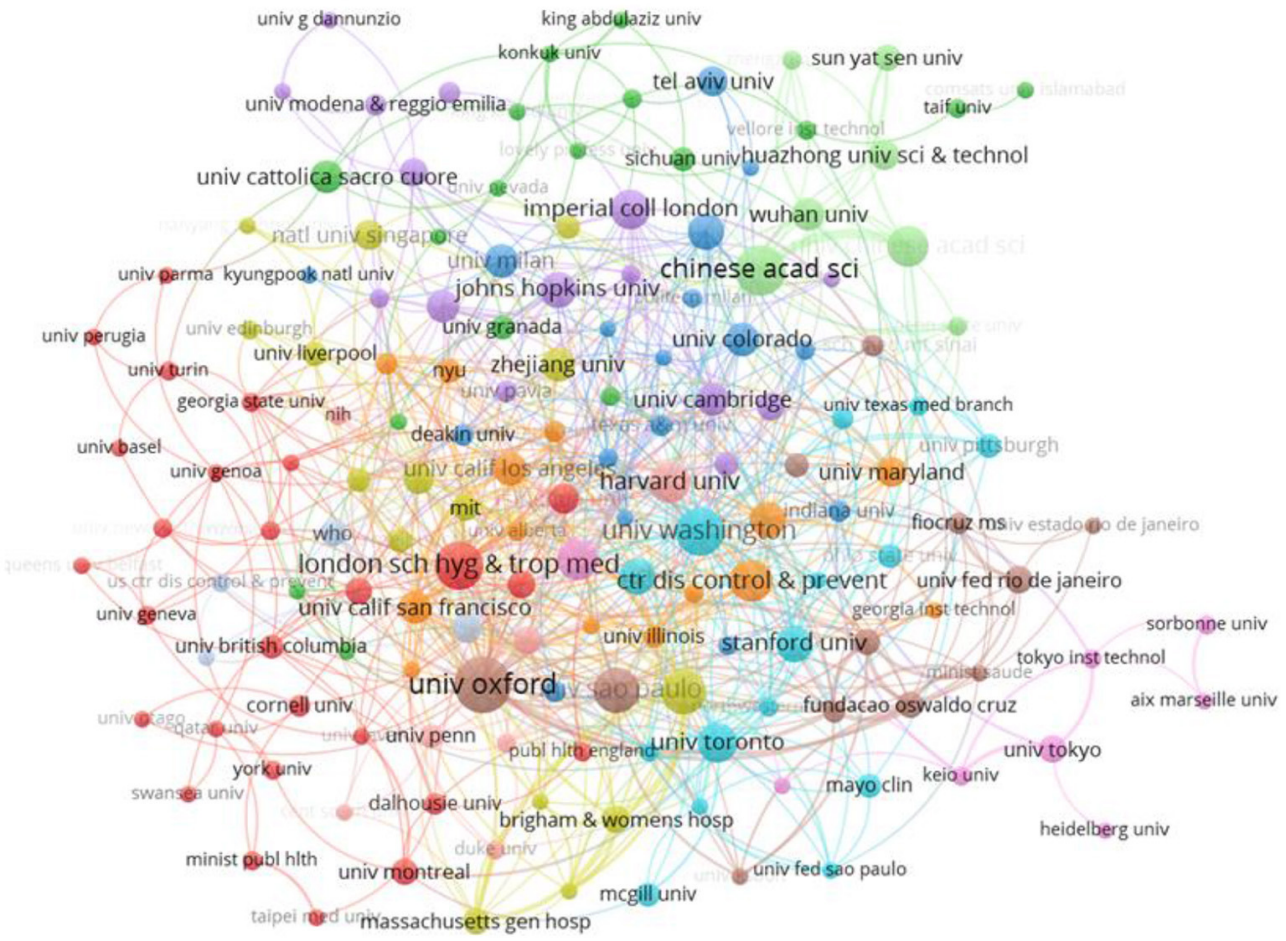
Knowledge domain map for co-authoring institutions.

The analysis of collaborative networks among institutions allowed the identification of institutions with high publication volume as well as finding institutions with an impact on smart emergency management of public health emergencies. To identify essential research institutions, we mapped the knowledge domain of collaborating institutions using VOSviewer software (Figure 4). The top 15 institutions that published papers were listed in Table 1. After the data were counted, a total of 3,621 research institutions were included in this study on emergency management of public health emergencies driven by digital technologies. Among the institutions that made it to the top 15, there were seven institutions in the United States, three in China, three in the United Kingdom, and one in Brazil and Canada. The University of Oxford in the UK published the most papers and had the largest TLS, indicating extensive collaboration. For CPP and H, CAS ranked first, showing a high academic impact in this area. The knowledge map of collaboration between institutions is in Figure 4. Each node represents an institution, the size indicates the number of articles issued, and the color represents the same cluster. The thickness of the connecting line indicates the strength of collaboration between the two institutions. The University of Oxford is the largest node, with strong cooperation with institutions such as the University of Washington, the London School of Hygiene and Tropical Medicine, Imperial College, and the University of São Paulo, and frequent collaboration with other national institutions. The second largest group of collaborating institutions is the Green Cluster. It mainly includes the Chinese Academy of Sciences, the University of Chinese Academy of Sciences, Wuhan University, and Huazhong University of Science and Technology. It also indicates that China's top research institutions closely cooperate with Chinese universities.

**Table 1:** Top 15 institutions with the most publication.

**Rank**	**Institution**	**Country**	**TD**	**TC**	**CPP**	**TLS**	**H**
1	Univ Oxford	England	27	976	37	60	11
2	Chinese Acad Sci	China	23	1439	63	34	13
3	Univ Washington	USA	21	690	33	46	9
4	London Sch Hyg and Trop Med	England	21	420	20	33	10
5	Univ São Paulo	Brazil	20	504	26	21	7
6	Harvard Med Sch	USA	19	522	28	45	8
7	Ctr Dis Control and Prevent	USA	18	574	32	21	10
8	Harvard Univ	USA	17	86	6	36	13
9	Univ Chinese Acad Sci	China	17	1142	68	24	9
10	Univ Hong Kong	China	17	377	23	10	8
11	Univ Toronto	Canada	16	1157	73	23	11
12	Imperial Coll London	England	16	654	41	19	8
13	Emory Univ	USA	14	655	47	26	8
14	Johns Hopkins Univ	USA	14	365	27	21	8
15	Stanford Univ	USA	14	223	16	19	6

TD, total documents; TC, total citations; CPP, citations per paper; TLS, total link strength; H, H-index.

### 3.4. Collaboration between authors

**Figure 5:**
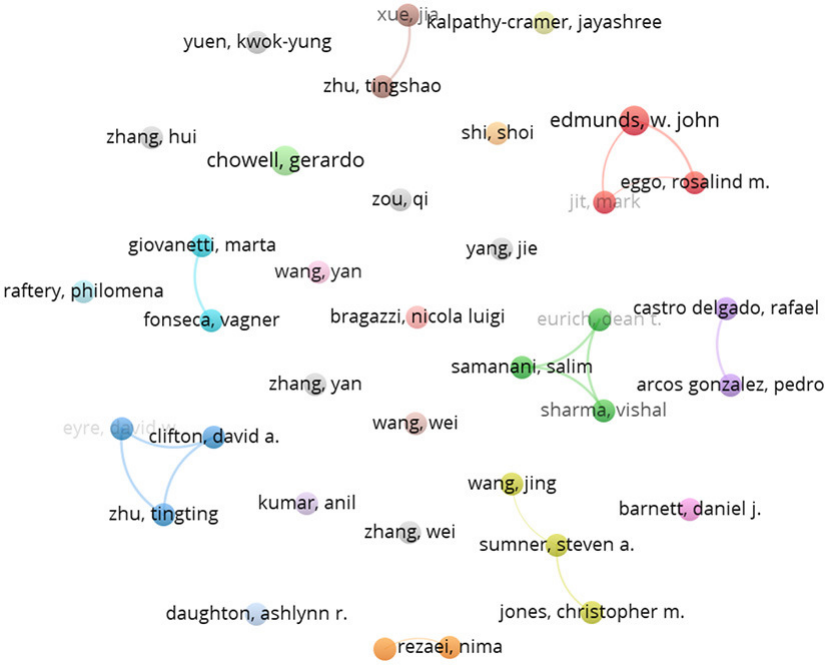
Mapping knowledge domains of co-author.

The analysis of author collaboration patterns in a field is useful for tracking the research trends and development directions in the field (44). As shown in Table 2, this paper lists the top 15 authors who have published the most articles in this research area. Prof. Rezaei, Nima from Tehran Medical University, Prof. Fonseca, Vagner from the University of KwaZulu-Natal, and Prof. Giovanetti from the University of Biomedical Sciences in Rome all published five articles. Edmunds, w.john from the London School of Hygiene and Tropical Medicine published four articles, and all other authors published three. In terms of the TC and CPP, Prof. Xue. Jia from the University of Toronto and Prof. Zhu Tingshao from the Chinese Academy of Sciences published the largest number of citations. In terms of total link strength (TLS), Clifton, David, Eire, David, and Zhu, Tingting of Oxford University have the highest number of co-authorship links with other authors, indicating that they collaborate extensively, and this can also be found in Figure 5. Among these authors, 5 of the top 15 are from the UK. Still, regarding academic impact (mainly referring to the H-index), Prof. Rezaei Nima from Tehran University of Medical Sciences has the highest academic impact with 4. Prof. Rezaei Nima has a long-term interest in digital technology in predicting, diagnosing, and treating autoimmune diseases in public health emergencies (45, 46).

**Table 2:** Top 15 authors with the most publication.

**Author**	**Country**	**Institution**	**TD**	**TC**	**CPP**	**TLS**	**H**
Xue, Jia	Canada	Univ Toronto	3	845	282	3	2
Zhu, Tingshao	China	Chinese Acad Sci	3	845	282	3	2
Edmunds,w.john	England	London Sch Hyg and Trop Med	4	77	20	5	3
Eggo.Rosalind.	England	London Sch Hyg and Trop Med	3	77	26	4	3
Clifton, David a.	England	Univ Oxford	3	34	12	6	2
Eyre, David w.	England	Univ Oxford	3	34	12	6	2
Zhu, Tingting	England	Univ Oxford	3	34	12	6	2
Rezaei, Nima	Iran	Univ Tehran Med Sci	5	33	11	1	4
Wang, jing	China	Nanjing Med Univ	3	23	8	1	
Fonseca, Vagner	South Africa	Univ Kwazulu natal	5	21	7	3	3
Giovanetti, Marta	Italy	Univ Campus Biomed Roma	5	21	7	3	3
Rzymski, Piotr	Poland	Poznan Univ Med Sciences	3	20	7	1	
Jit, Mark	China	Univ Hong Kong	3	6	2	3	2
Sumner, steven.	USA	Ctr Dis Control and Prevent	3	6	2	3	1

TD, total documents; TC, total citations; CPP, citations per paper; TLS, total link strength; H, H-index.

### 3.5. Analysis of research hotspots and evolutionary trends

#### 3.5.1. Co-occurrence analysis of keywords

**Figure 6:**
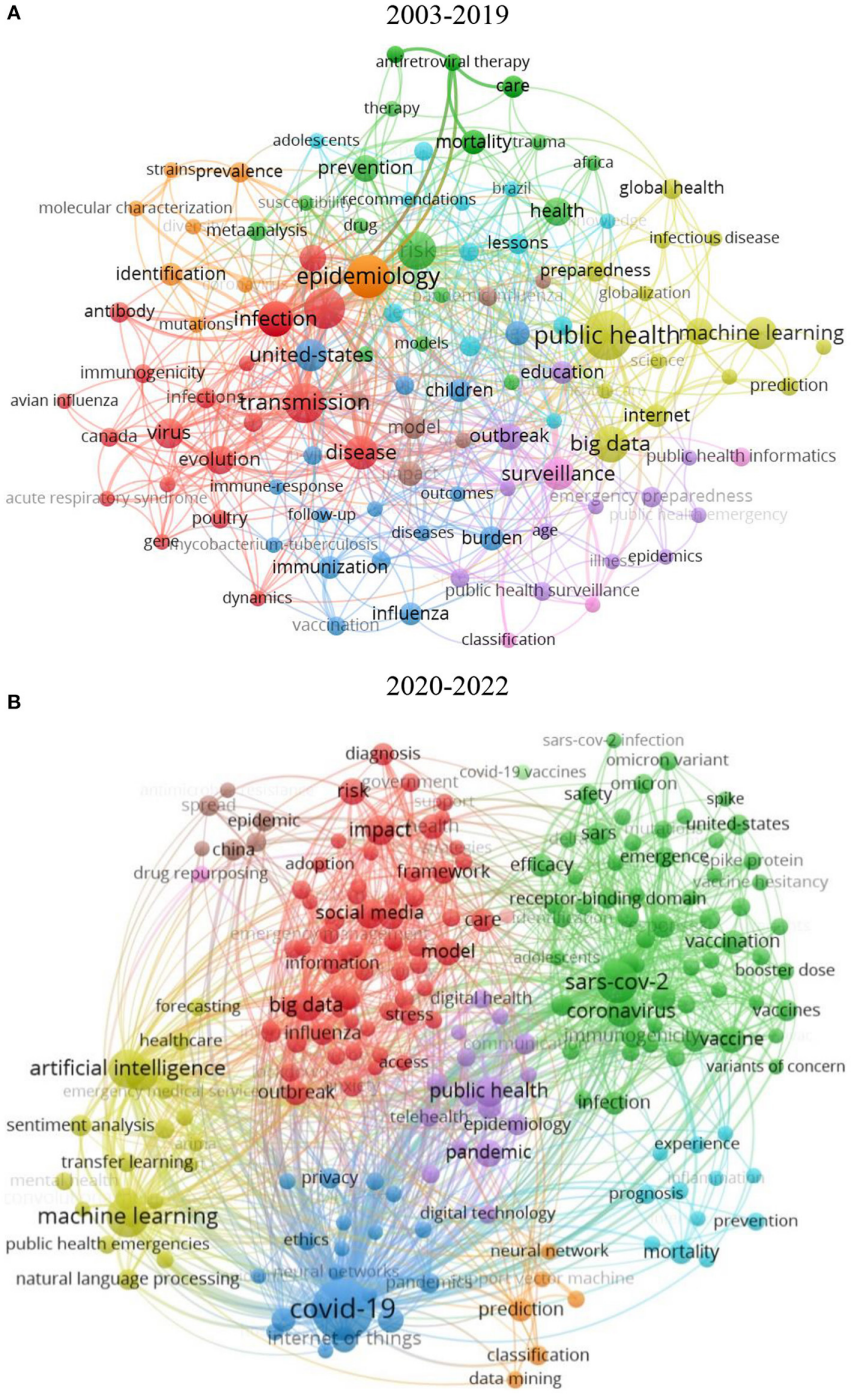
**(A)** Mapping knowledge domains of the keywords plus co-occurrence network from 2003 to 2019. **(B)** The keywords plus co-occurrence network from 2020 to 2022.

As the core of academic research and the label of disciplinary information, keywords can highly summarize the main content of an article and can reveal the main content features and research directions of a paper (47–49). Cluster analysis can reveal the close relationship between keywords and their degree of relevance (50). It has been shown that author keywords have the defects of high subjectivity and poor statistical properties, while Plus keywords obtained from WOS tend to reduce the interference of subjectivity and have higher accuracy and statistical value. Therefore, this part focuses on Plus Keywords as the research object and uses VOSviewer for cluster analysis. The visual analysis of the drawn knowledge graph is shown in Figure 6. Each node represents a keyword, and the size of the node represents the frequency of occurrence in the article. The thickness of the connecting lines presents the co-occurrence strength between the keywords.

In Figure 6A, cluster 1 (red) is mainly centered on the keyword “transmission,” which has the largest number of occurrences and TLS in cluster 1. The keyword “transmission” is closely related to the core keywords “epidemiology” and “risk” in Cluster 2 (orange) and Cluster 3 (green). The three clusters have a high level of cross-fertilization, indicating a focus on the risk impact of epidemic transmission at this stage. The second largest cluster 4 (gold) is centered on the keyword “public health,” with the keywords “machine learning” and big data. The keywords “machine learning,” “big data,” and “prediction” are closely related to their core, indicates that machine learning and big data methods are mainly applied to the prediction of public health events and provide prevention and emergency preparedness solutions for major disease outbreaks.

In Figure 6B, cluster 1 (green) is mainly centered on the keyword “Sar-Cov-2.” Cluster 1 also includes the keywords “vaccination,” “vaccine,” “coronavirus,” and “omicron variant,” indicating that variation, vaccination, and vaccination of COVID-19 are essential issues for scholars. Cluster 2 (gold) has a high co-occurrence with cluster 3 (red) and cluster 4 (blue). The high co-occurrence of the keywords “artificial intelligence,” “machine learning,” “natural language processing,” “transfer learning,” and “big data” indicates that the extensive use of digital technology resources by scholars to contain the spread of new coronaviruses under the COVID-19 epidemic. To detect, monitor, and manage outbreaks as well as to perform data analysis and decision-making processes, a large number of scholars used artificial intelligence-driven machine learning, deep learning, transfer learning, or natural language processing and Internet of Things (IoT)-enabled blockchain technologies and big data to research (51).

#### 3.5.2. Cluster analysis based on a two-mode matrix

**Figure 7:**
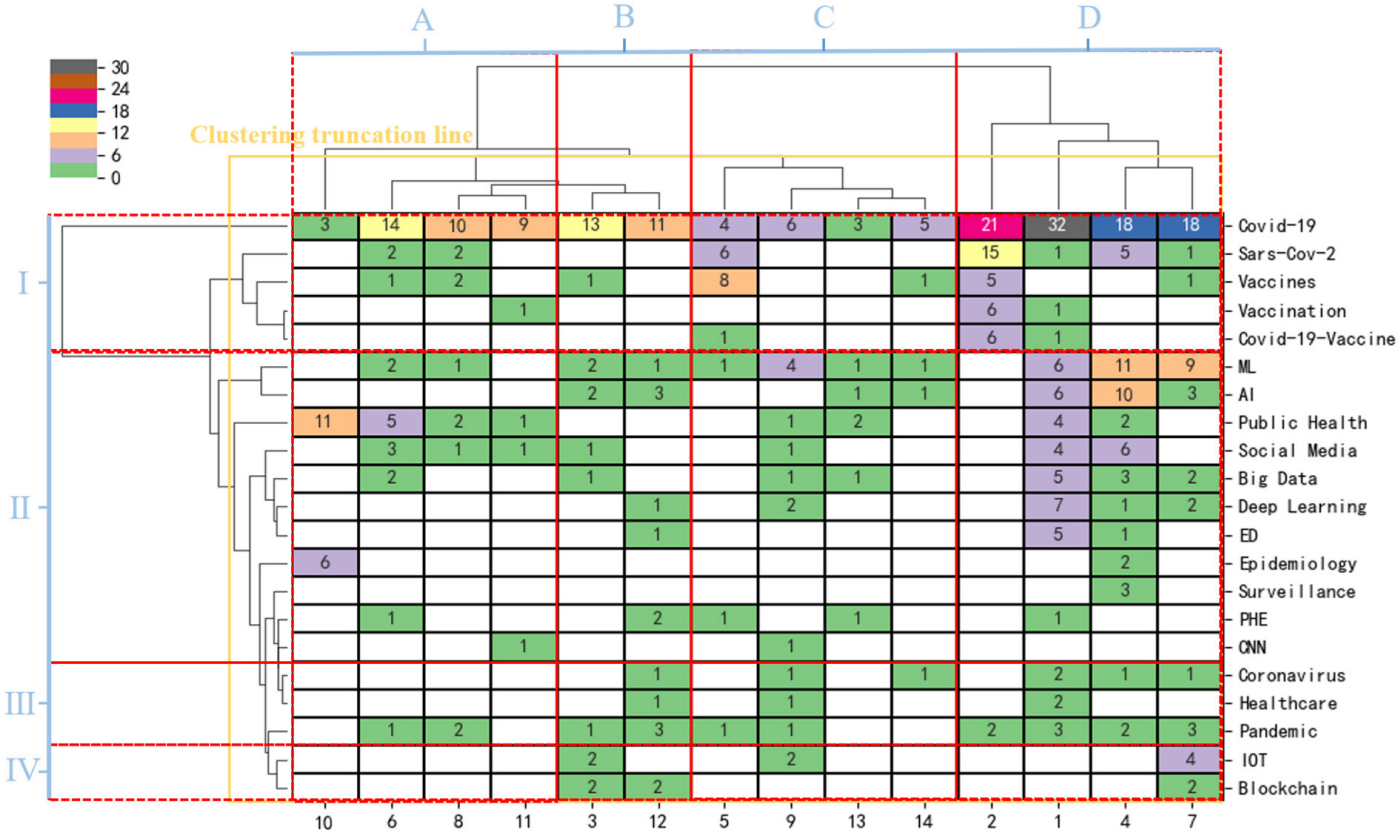
Mapping knowledge domain of system clustering pedigree tree based on the two-module matrix of journals-keywords. ML, machine learning; AI, artificial intelligence; ED, emergency department; PHE, public health emergency; CNN, convolutional neural network; IOT, internet of things.

Cluster analysis can directly show the close relationship between keywords and identify their degree of relevance. Examining only one dimension of clustering may ignore the influence of the partnership of other dimensions (e.g., journals, institutions, and authors) on clustering. Therefore, we used COOC software to construct a two-mode matrix and perform a hierarchical clustering analysis of high-frequency journals and keywords. Figure 7 shows the bidirectional clustering results for emergency management of public health emergencies driven by digital technology. The horizontal clustering tree represents the clustering results of journals, and the vertical clustering tree represents the clustering results of keywords. The right side of Figure 7 shows 21 high-frequency keywords, and the bottom shows 14 high-frequency journals.

Through hierarchical cluster analysis, we found that the hotspots are mainly concentrated in four parts: (i) research on vaccines for new coronaviruses and vaccination, mainly including COVID-19-Vaccine, Vaccination; (ii) research on intelligent management of public health emergencies, mainly machine learning, artificial intelligence, big data, deep learning, social media, convolutional neural network, and supervised learning in public health emergencies; (iii) research on the spread of new coronaviruses and healthcare, mainly Healthcare, Pandemic; and (iv) research on the Internet of Things and blockchain technology in public health emergencies, with the main subject terms IoT, Blockchain. High-frequency journals are also divided into four research areas according to their relevance. (A) Frontiers in Public Health, Frontiers in Psychology, and Epidemiology clusters; (B) Sustainability, Healthcare, and Vaccines clusters; (C) Applied Sciences, Disaster Medicine and Public Health Preparedness, and Environmental Sciences clusters; and (D) Vaccines, International Environment and Public Health, Internet of Medicine, and Information Technology and Engineering citation clusters. Categories (C) and (D) can be further classified into one category, indicating that information and intelligent management are the general trends of future research on public health emergency management. It is worth noting that (i) the COVID-19 vaccines and vaccination are currently popular research directions in this field, while (C) and (D) are the most popular published journals, especially nodes (D)-(i). (ii) Artificial intelligence, machine learning, and big data have been critical technical tools for public health emergency research in recent years. The research shows a rising trend.

### 3.6. Research frontier identification

**Figure 8:**
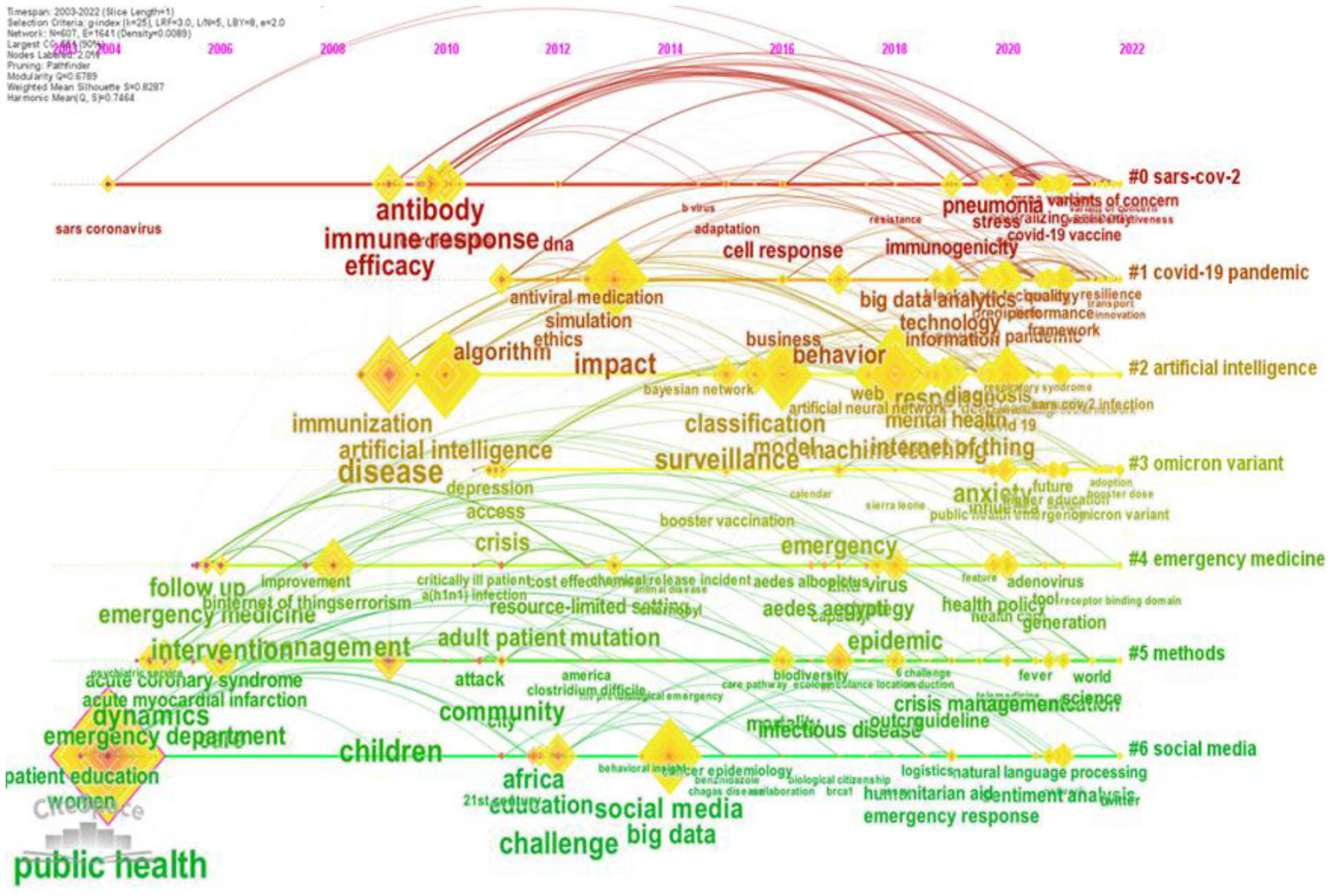
The keywords view of publications on emergency public health emergency management from 2003 to 2022.

In order to explore the frontier research on the management of public health emergencies driven by digital technologies, it is necessary to study the timeline evolution of publication keywords. Therefore, we plot the keyword event line visualization in the field of public health emergencies, as shown in Figure 8. The keyword timeline shows the research progress over time for eight clusters that can be used to assess research topics. The *x*-axis represents the year of publication, and the *y*-axis represents the clusters of keywords. Each cluster contains multiple keywords; the larger the node of a keyword, the more frequently it appears. The node linkage represents the co-occurrence relationship of the keywords.

Cluster #0 (SARS-CoV-2) contains the keywords antibody, immune response, pneumonia, immunogenicity, vaccine, and efficacy. SARS-CoV-2 is the abbreviation for severe acute respiratory syndrome virus 2. Vaccination effectively prevents and controls COVID-19 infection, through which the body produces antibodies and becomes immune (52). However, vaccination-induced immunogenicity varies from person to person. Only a full understanding of vaccine immunogenicity and other factors can provide a targeted immunization strategy based on human variability. Therefore, in a major public health emergency such as COVID-19, the focus is on vaccine research and immune responsiveness.

Cluster #1 (COVID-19 pandemic) contains keywords such as impact, algorithm, big data analytics, blockchain technology, behavior, emergency management, and decision making. Big data, behavioral analytics, and blockchain digital technology provide intelligent decision-making for emergency management of public health emergencies. These digital technologies can be used to screen, detect, monitor, regulate, and track the management of coronaviruses to control the spread of coronavirus infections and contain pandemics (53, 54).

Cluster #2 (artificial intelligence) mainly includes disease, artificial intelligence, classification, surveillance, machine learning, deep learning, the internet of things, recognition, and other keywords. From 2003 to 2019, some scholars mainly used artificial intelligence techniques and some models for the classification and surveillance of sudden public diseases. And the medical decision support systems were applied to clinical diagnosis. After 2020, all kinds of artificial digital technologies are utilized, ranging from intelligence-driven machine learning (ML) or deep learning (DL) focused applications to blockchain technologies enabled by the internet of things, becoming essential tools for researchers to identify and diagnose major sudden public diseases (51). Random forests and convolutional neural networks in machine learning are the more applied methods in public health emergencies.

Cluster #0 (SARS-CoV-2) and Cluster #1 (COVID-19 pandemic) Cluster #3 (Omicron variant) can be grouped in essence, and COVID-19 caused by SARS-CoV-2 poses a health threat to the global population. Global efforts to determine the origin, structure, and pathogenesis of SARS-CoV-2 variants have explicitly focused on variants of interest, such as the Delta Variant and Omicron Variant (55). Omicron Variant is applicable to a range of non-pharmaceutical interventions targeting SARS-CoV-2. The development of vaccines for both COVID-19 and SARS-CoV-2 could help control Omicron's spread and infection, a popular topic of scholarly research (56–58).

Cluster #4 (emergency medicine) mainly includes keywords such as management, epidemic, strategy, adult patient, health policy, and health care. Cluster #5 (methods) mainly includes keywords such as emergency department, care, children, infectious disease, and mortality. Cluster #4 and cluster #5 can be grouped, and the COVID-19 pandemic exposed the global need for public health departments and emergency departments to develop strategies to address complex public health challenges, which require enhanced public health education (59, 60). In addition, public health policies and health care education are essential strategies to respond to public health crises (59). Therefore, they tend to be topics of interest to researchers during outbreaks. In the last decade, infectious diseases under public health emergencies have been a major threat to children's health in developing countries, which has prompted some scholars to focus on the research on the impact of public health interventions (promotion, protection, prevention) on morbidity and mortality caused by infectious diseases in children (61, 62).

Cluster #6 (social media) mainly includes keywords such as public health, big data, Twitter, emergency response, sentiment analysis, and natural language processing. Major public health events are usually characterized by suddenness, complexity, uncertainty, and danger (3). Government emergency management departments can effectively collect big data from social media such as Weibo, WeChat, and Twitter and use specialized tools and methods (natural language processing, sentiment analysis, and text analysis) for data mining and feature extraction of information related to different topics, sentiments, and emotions (63–65). In turn, governments can respond quickly to achieve optimal public health decisions, thereby mitigating the adverse effects of public health events and promptly returning to normalcy (3).

Burst detection is a more-than-citation-counting method for identifying topics that have received significant attention at different stages of development. The top 25 burst keywords, along with the burst intensity and duration, are shown in Table 3. The burst keywords shown in Table 3 are consistent with those clustered above.

**Table 3:** Top 25 keywords with the strongest citation bursts.

**Keywords**	**Year**	**Strength**	**Begin**	**End**	**2003– 2022**
Virus	2004	4.07	2004	2016	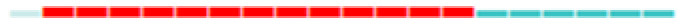
Burden	2008	4.41	2008	2018	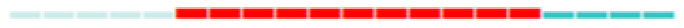
Antiretroviral therapy	2009	3.11	2009	2019	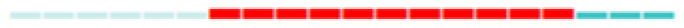
Immunization	2009	2.59	2009	2019	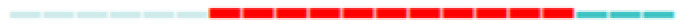
Children	2009	2.54	2009	2011	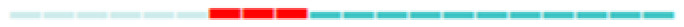
Public health	2004	10.39	2011	2019	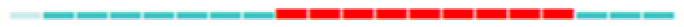
Emergency	2009	8.11	2012	2019	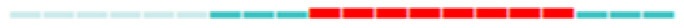
Africa	2012	2.33	2012	2018	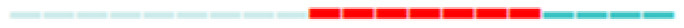
Epidemiology	2013	7.61	2013	2019	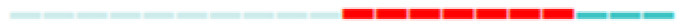
Disease	2009	7.11	2013	2018	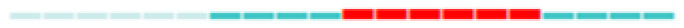
Transmission	2004	3.66	2013	2019	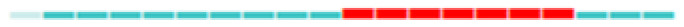
Evolution	2004	2.59	2013	2019	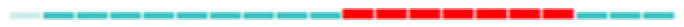
United States	2014	4.16	2014	2018	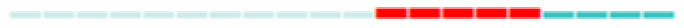
Big data	2014	5.09	2015	2020	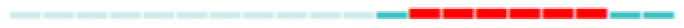
Risk	2011	3.6	2015	2017	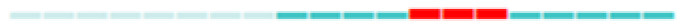
Surveillance	2015	2.41	2015	2019	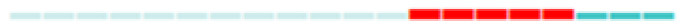
Outbreak	2011	3.82	2016	2019	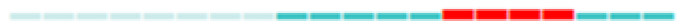
Infection	2005	2.52	2016	2019	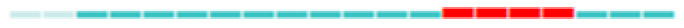
Infectious disease	2017	4.97	2017	2020	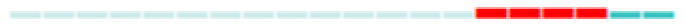
Emergency	2017	2.37	2017	2018	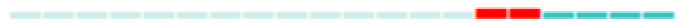
Zika virus	2018	3.14	2018	2019	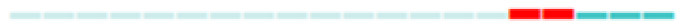
Mental health	2019	2.89	2019	2020	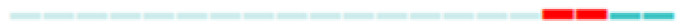
Influenza	2020	4.12	2020	2022	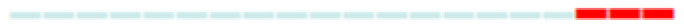
Neural network	2020	3.89	2020	2022	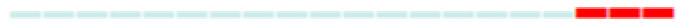
Pneumonia	2020	2.74	2020	2022	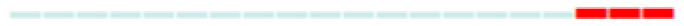

## 4. Discussion

In recent years, the frequency and complexity of public health emergencies have increased with the rapid advancement of globalization (49). With the catalyst of the new coronavirus pneumonia pandemic, digital technologies have been rapidly developed, and artificial intelligence, machine learning, deep learning, big data, blockchain, and Internet of Things technologies have been widely applied to the prevention and control of infectious diseases, enhancing the ability of public health systems to deal with emergencies and normalize prevention and control (66, 67). Digital technologies provide preventive preparedness, surveillance and early warning, and emergency intelligent response management for the whole life cycle of public health emergencies (68) but also can guide countries in formulating health policies and improve the ability to respond to public health emergencies together globally.

Research on emergency management of public health emergencies driven by digital technologies made continuous progress from 2003 to 2020, which can be divided into the growth and development period (2003–2019) and the rapid development period (2020–2022). During the growth and development period, the utilization of relevant digital technologies has focused on advanced clinical decision support systems with artificial intelligence and medical sensor integration to help decision-makers and healthcare systems improve the way they process information (69). After 2019, the published papers show a diversified incremental due to the outbreak of the global public health emergencies of the COVID-19 epidemic. The breadth and depth of the application of digital technology in the emergency management of the COVID-19 epidemic has been significantly enhanced (51, 70, 71). Regarding the volume of publications in the literature, the country with the highest number of published papers is the United States, accounting for 17.6% of the total literature. The USA carried out earlier applications of digital technologies in emergency management of public health events, with a more significant impact in this field. China ranked second in terms of the number of papers published. And the application of digital technology in healthcare has developed rapidly in recent years, with an extensive fund investment in epidemic prevention and control. In terms of institutional cooperation, the University of Oxford and the Chinese Academy of Sciences are the core institutions with an essential link in the paper publication network. Regarding academic influences, Prof. Rezaei, Nima from the Medical University of Hyland, Germany, has an H-index of 4. He has achieved significant scientific results in predicting, diagnosing, and treating autoimmune diseases (45, 46).

In terms of research hotspots and evolutionary trends, with the continuous development of digital technology, the application of big data analysis, machine learning, blockchain technology, and the Internet of Things has become a hotspot for research on emergency management of public health emergencies. The information and intelligent management of public health events is a major trend in future emergency management research of public health emergencies (1, 65). It is worth noting that the research on the application of digital technology in the vaccination and vaccination of new coronavirus is also a hot spot for research (72). In addition, based on the cluster analysis of literature, four core journal clusters of digital technology in emergency management of public health emergencies were summarized. The systematic clustering results of the bimodal matrix indicated that the frontiers of public health journal (A), Sustainability journal (B), applied science journal (C), and international environmental research and public health journal (D) have strong co-citation relationships. And they are the dominant directions for future research in this field.

In terms of timeline analysis and research frontier identification, bibliometric methods were used to identify “Sar-Cov-2,” “COVID-19 pandemic,” “artificial intelligence,” “omicron variant,” “emergency medicine,” “social methods,” “social media,” and other seven categories. The categories “COVID-19 pandemic” and “omicron variant” both occurred in 2020 and included the keywords immunogenicity and vaccine, indicating the origin, structure, pathogenesis, and diagnostic and therapeutic issues of new coronaviruses are still at the forefront of research on global public health events (55). Meanwhile, focusing on public health policy and health care education is an essential strategy for responding to public health crisis emergencies in the future (60, 65). The utilization of social media data collected from microblogs, WeChat, and Twitter by emergency management departments to mine the information features of public health events to promote digital and smart governance in public health events will be a frontier issue that must be paid attention continually to currently and even in the future (3, 63, 64).

## 5. Limitations

The limitation of this research is that only representative WOS literature databases are selected. Still, the number of articles included in this database is limited, and databases such as Scopus, PubMed, and Willey are omitted. Meanwhile, the selection of literature in this paper focuses on published English literature, and relevant literature in other languages may be lost. In addition, this paper does not consider semantic normalization of the collected data, which would affect understanding the semantic content. Finally, this study chose to analyze the literature on emergency management of public health emergencies driven by digital technologies from 2003 to 2022 and did not filter the retrieved journals by high citation rate and impact factor. Therefore, the quality of the articles selected for the study could be further improved.

## 6. Conclusions and future works

The research on emergency management of public health emergencies driven by digital technologies is numerous and complex. Therefore, having a clear and comprehensive understanding of the rapidly growing scientific literature information is difficult. VOSvieewer, Citespace, and COOC software were utilized to visually analyze 1,644 articles in the Web of Science core database. Then this study sorted out emergency management strategies and frameworks for emergency management of public health driven by digital technologies and showed how the relevant research evolved in this field between 2003 and 2022. The relevant research directions and conclusions are as follows.

First, there is an imbalance in the regional distribution of scientific research power in emergency management of global public health emergencies driven by digital technology. The USA, China, the United Kingdom, Italy, and India are the top five countries in the global digital emergency management of public health emergencies, accounting for 77.6% of the total literature. And the level of cooperation among various countries and institutions is generally low, the number of highly productive authors is low, and there are fewer teamwork studies across regions.

Second, in the face of uncertainty and sudden public health events, digital technologies consisting of artificial intelligence, big data analysis, machine learning, artificial intelligence, the Internet of Things, and blockchain will promote intelligent and informative management of public health events. However, digital public health does not completely replace traditional public health practices. Still, it improves upon them, increasing the coverage of public health services, improving service effectiveness, and reducing costs. The application of digital technology in public health emergency management is still in the initial development stage. Many problems, such as data gaps, data silos, and lack of data cases, need to be solved. In particular, the COVID-19 pandemic exposed the vulnerability of the global emergency management cooperation mechanism for public health emergencies. Therefore, in order to promote intelligent response and smart decision-making capabilities for major global public health emergencies in the future, countries must strengthen the exchange of healthcare-related digital technologies and promote the construction of digital public health emergency management systems. Eventually, an efficient monitoring and early warning and intelligent emergency protection system for the whole life cycle of public health emergencies will be established.

## Data availability statement

The original contributions presented in the study are included in the article/supplementary material, further inquiries can be directed to the corresponding authors.

## Author contributions

Conceptualization: CW and WL. Methodology, software, and visualization: WL, CL, and ZH. Formal analysis: WL and CL. Investigation, writing—original draft preparation, and funding acquisition: CW. Resources and project administration: ZH. Data curation and supervision: CL. Writing—review and editing: WL and ZH. All authors have read and agreed to the published version of the manuscript.
